# Functional MgAl LDH@SiO_2_ Composites: Controlled Fluoride Delivery in Dentistry

**DOI:** 10.3390/molecules31122180

**Published:** 2026-06-22

**Authors:** Asma Alazreg, Marija M. Vuksanović, Vladisav Tadić, Adela Egelja, Andrija Savić, Aleksandra Šaponjić, Radmila Jančić Heinemann

**Affiliations:** 1 Faculty of Technology and Metallurgy, University of Belgrade, 11000 Belgrade, Serbia; 20194028@estudent.tmf.bg.ac.rs; 2 Department of Chemical Dynamics and Permanent Education, “VINČA” Institute of Nuclear Sciences-National Institute of the Republic of Serbia, University of Belgrade, 11351 Belgrade, Serbia; marija.vuksanovic@vin.bg.ac.rs (M.M.V.); adela@vin.bg.ac.rs (A.E.); savic@vin.bg.ac.rs (A.S.); 3 Institute of Chemistry, Technology and Metallurgy, National Institute of the Republic of Serbia, University of Belgrade, 11000 Belgrade, Serbia; vladisav.tadic@ihtm.bg.ac.rs; 4 Department of Materials, “VINČA” Institute of Nuclear Sciences-National Institute of the Republic of Serbia, University of Belgrade, 11351 Belgrade, Serbia; acavuc@vin.bg.ac.rs

**Keywords:** layered double hydroxide, silica particles, fluoride release, composite materials

## Abstract

Bio-silica particles derived from rice husks were coated with MgAl layered double hydroxides (LDHs) and thermally converted into layered double oxides (LDOs) to evaluate fluoride capture and release capability. The deposition of an MgAl LDH layer on the silica particle makes the LDH more accessible for interaction. Fluoride loading was tested in aqueous and ethanol–water media, with mixed solvents consistently enhancing uptake. Release studies in demineralized water showed relatively rapid desorption (~1500 min), whereas embedding particles in an acrylic matrix reduced the release rate by nearly two orders of magnitude, enabling sustained release levels suitable for dental applications. Ethanol promoted both ion exchange and memory effect mechanisms, providing tunable control over fluoride incorporation and release. These functional composites demonstrate potential for controlled delivery in dental restorative materials, highlighting their potential as adaptive fillers that can enhance the mechanical properties while also serving a functional base for low fluoride release.

## 1. Introduction

Layered double hydroxides (LDHs) are versatile materials used in anion removal, catalysis, and controlled release [1]. Their ion exchange capacity enables dynamic adaptation to chemical environments. When heated to ~450 °C, LDHs dehydrate and transform into layered double oxides (LDOs), expelling interlayer anions and collapsing the lamellar structure [2]. LDOs exhibit a “memory effect”: upon rehydration with suitable anions, they reconstruct the LDH framework, allowing complete substitution of interlayer species. This reversible transformation restores the layered configuration and enhances functional tunability.

Silica (SiO_2_), one of the most abundant minerals in Earth’s crust, is a key component of many rock types [2,3]. It also plays important biological roles: plants such as rice incorporate silica to strengthen tissues and resist stress [4,5]. Rice husks are particularly rich in bio-silica, which is highly pure and finely structured [6,7]. After acid leaching and thermal processing, this agricultural waste yields amorphous silica with high surface area, thermal stability, and mechanical strength [7]. Bio-silica can be integrated with LDHs to form hybrid structures with applications in dentistry, catalysis, and remediation [8,9]. Such composite particles, when added to acrylic composites for denture preparation as reinforcements, improve mechanical properties [10]. Multication crystal systems further expand functional possibilities [11].

Fluoride is essential for enamel formation and long-term dental health. Systemic intake during childhood promotes fluorapatite formation, a mineral more acid-resistant than hydroxyapatite, while topical application is essential [12]. Deficiency increases susceptibility to caries [13], whereas excessive exposure may cause dental fluorosis [14] or, in severe cases, skeletal fluorosis [15,16].

Advances in adhesive dentistry introduced acrylate-based restorative materials with strong mechanical and aesthetic properties [17]. Photopolymerization further improves their use [18]. Incorporating fluoride-releasing fillers into composites enables localized delivery, enhancing enamel resistance [19,20]. Sustained release strategies include fluoride-bearing fillers and surface-modified nanostructures for controlled fluoride release [18,21].

Deposition of a layer of LDH on the bio-silica fine particles enhances the surface of the available material that is open to fluoride capture, but it also opens the possibility of containing a larger amount of fluoride and thus creating a better reservoir for fluoride for dental material. On one hand, the layered double hydroxide and oxide structures demonstrate high affinity for fluoride ions, enabling efficient capture from contaminated water sources and offering a sustainable solution for communities facing fluoride-related health risks. On the other hand, the same structural features, particularly the ion exchange capacity and memory effect reconstruction, allow controlled release of fluoride when embedded in dental composites, providing localized and sustained delivery that strengthens enamel and reduces caries risk. This dual utility exemplifies the growing paradigm in materials science toward adaptive, multifunctional materials that expand the possibilities of use in environmental and healthcare domains. By combining bio-derived silica with tunable LDH/LDO coatings, the present work introduces a platform material that not only advances dental restorative technology but also contributes to broader efforts in sustainable water treatment, underscoring its relevance to the wider materials community [22].

The aim of this study was to develop a multifunctional reinforcement for dental composites that opens the possibility of controlled fluoride release, improved mechanical properties, and color compatibility. Fluoride charging was examined through ion exchange and memory effect mechanisms using silica particles coated with LDH or thermally converted LDO. The effect of solvent composition was also evaluated, comparing water with ethanol–water mixtures. Release behavior was assessed in composite particles where silica cores were coated with LDH and transformed into LDO after heat treatment. The particles, after being exposed to NaF solution in water and ethanol–water mixtures, were examined. Particles were introduced into an acrylic matrix, where they served both as modifiers and functional fillers. Fluoride release was monitored for the particles and for the acrylic matrix filled with the same particles.

## 2. Results and Discussion

### 2.1. Structural Analysis of Samples

X-ray diffraction (XRD) was employed to examine the structural characteristics of the composite particles. The silica, which underwent heat treatment at 800 °C during preparation, exhibited broad peaks indicative of the initial stages of crystalline phase formation.

**Figure 1:**
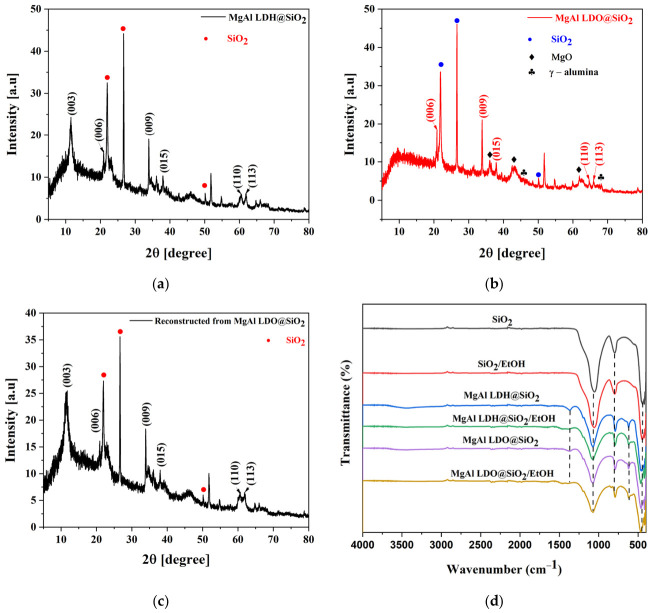
(**a**) XRD pattern of synthesized MgAl LDH@SiO_2_, (**b**) XRD pattern of synthesized MgAl LDO@SiO_2_, (**c**) the restructured sample of MgAl@SiO_2_ restructured from the MgAl LDH@SiO_2_ in water/ethanol solution, (**d**) FTIR analysis of specimens after immersion in water solution of fluoride and in water/ethanol solution of fluoride.

The MgAl LDH synthesized via precipitation showed a typical layered double hydroxide pattern. Peaks at 2θ ≈ 11°, 22°, 34°, 46° and 62° correspond to characteristic diffraction maxima for the LDH structure, confirming that the coprecipitation produced the layer of LDH on the substrate particles (Figure 1a). The maximum at exactly 2θ = 11.4° corresponds to the MgAl LDH structure and the gallery spacing of d ≈ 7.7 Å. The characteristic peak of silica at 21° and 26° illustrates the presence of silica in the structure. Analysis of the composite powder confirmed the presence of both the silica substrate and the MgAl LDH phase. Heat treatment at 450 °C destroyed the LDH structure, yielding LDO with a remnant layered framework capable of reconstructing into LDH via the memory effect when exposed to aqueous fluoride solutions. The diffractogram does not have the characteristic peak at 11° but peaks for MgO at 37°, 43° and 62° of low intensity indicate the degradation of LDH into the MgO structure; peaks at 46° and 60° are of low intensity and indicate the formation of low crystalline alumina phases (Figure 1b). The characteristic peaks of silica remain present in the structure.

The reconstruction was confirmed by XRD analysis of LDO samples treated in both water and ethanol–water NaF solutions. The same peaks as those initially obtained for coprecipitation-formed MgAl LDH@SiO_2_ are identified, but they are broader than in the coprecipitated hydroxide structure (see Figure 1c).

FTIR spectra of all specimens are shown in Figure 1d. The expected Si–O, Mg–O, and Al–O bonds were observed. The broad O–H stretching band at 3400–3600 cm^−1^ is faint in all structures [23], appearing slightly in LDH, consistent with interlayer water. Correspondingly, the H–O–H bending at 1650 cm^−1^ is present only in LDH and absent in oxide forms. A weak band at 1350–1500 cm^−1^, attributed to CO_3_^2−^ from synthesis under atmospheric conditions, is less pronounced in ethanol-treated samples. Mg–O and Al–O vibrations in the 400–800 cm^−1^ [24] range confirm the LDO structure [25], visible in both aqueous and ethanol preparations. Minor NO_3_^−^ traces remain from precursor salts. Silica features are evident in all SiO_2_ based samples: strong asymmetric Si–O–Si stretching at 1080–1100 cm^−1^ [26], symmetric stretching at 790–800 cm^−1^, and bending at 460 cm^−1^. The broadening of the 1080 cm^−1^ band reflects the amorphous nature of silica. Fluoride incorporation is not directly visible in FTIR, though slight shifts in Mg–O and Al–O bands suggest its influence.

**Figure 2:**
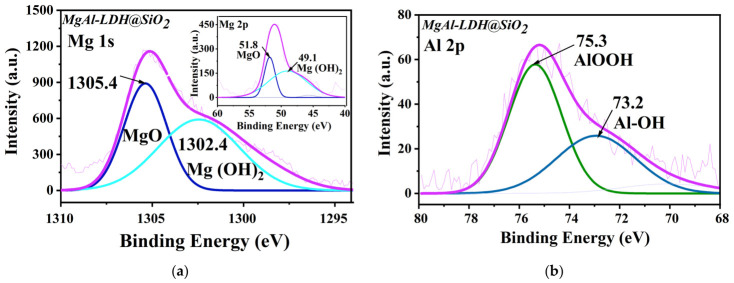

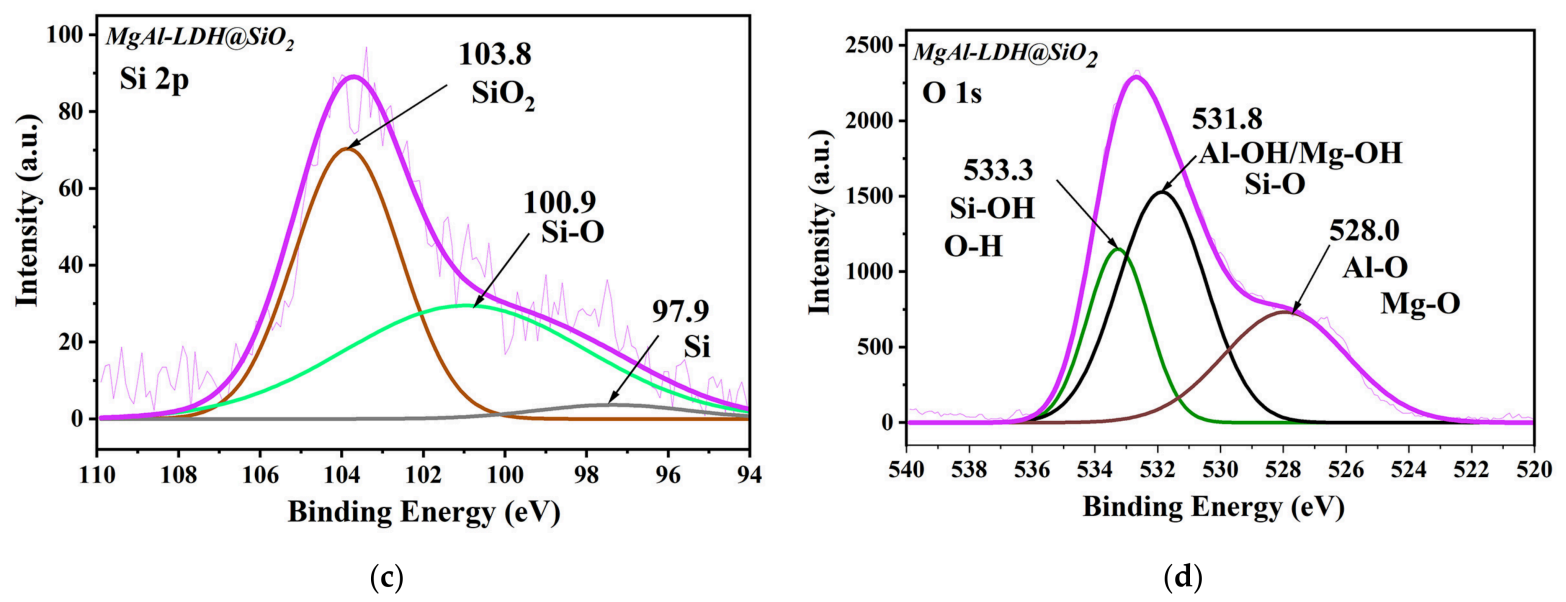
XPS spectra of Mg–Al–LDH@SiO_2_: (**a**) Mg, (**b**) Al, (**c**) Si, and (**d**) O core levels.

The survey spectrum of MgAl–LDH@SiO_2_ shows the presence of Mg, Al, Si, and O in the 1s, 2s, and 2p orbitals. The Mg 1s and Mg 2p spectra confirm Mg^2+^ states at 1302.4–1305.4 eV and 49.1–51.8 eV, while the Al 2p spectrum reveals Al^3+^ states at 73.2–75.3 eV, corresponding to Mg(Al)–OH/Mg(Al)–O [27]. The Si 2p spectrum is characterized by three deconvoluted peaks at 97.9, 100.9, and 103.8 eV, related to elemental Si and SiO_2_ [28]. The O 1s spectrum exhibits three components at 528.0, 531.8, and 533.3 eV, attributed to Mg(Al)–O, Mg(Al)–OH/Si–O, and Si–OH/adsorbed H_2_O [29] (Figure 2).

### 2.2. Morphology Images of Samples (TEM, SEM and EDS)

**Figure 3:**
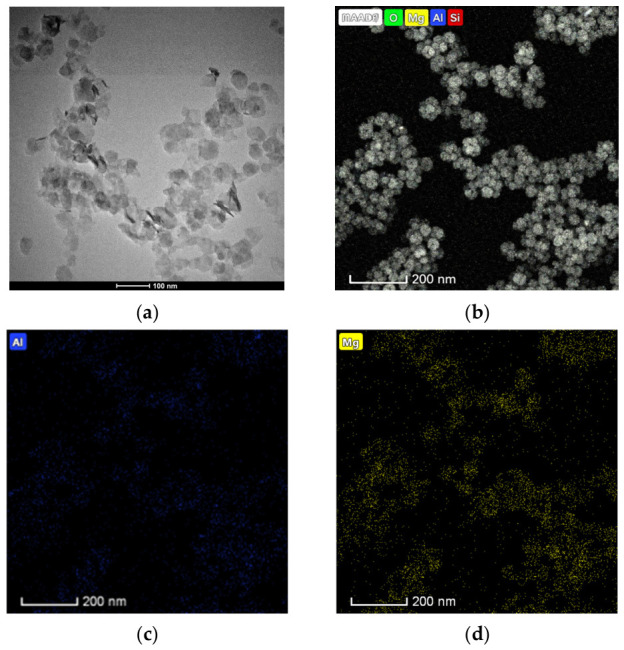

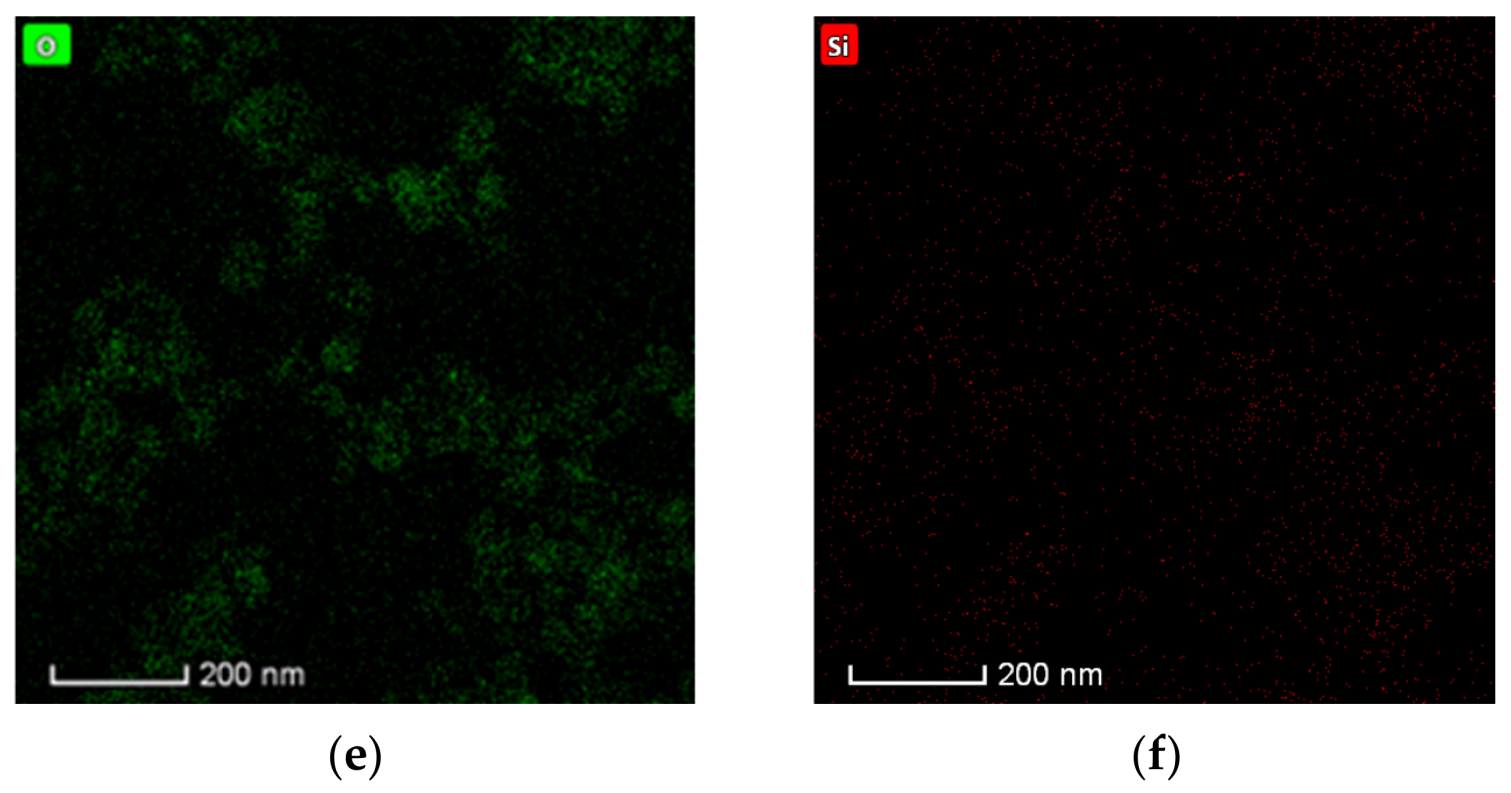
(**a**) TEM image of synthesized particles confirming their very fine structure; (**b**) the EDS of synthesized particles, confirming the uniform composition where atomic species of Si, Al, Mg, and O are thoroughly intermixed; (**c**) the placement of Al; (**d**) the placement of Mg; (**e**) the placement of O; and (**f**) the placement of Si.

The structure of the composite particles after synthesis was examined using the TEM imaging technique and to the sample the EDS was applied to verify the structure and placement of Si, Al, Mg and O atoms. The images confirm the very fine structure of particles, diameters under 100 nm, and the very close positioning of all Si, Al, Mg and O atoms in the structure, confirming that the coprecipitation took place on the surface of silica particles, creating the composite structure at this level. Figure 3a shows the TEM image of synthesized particles, confirming their fine structure, and Figure 3b exhibits the EDS image where the homogeneous placement of Si, Al, Mg and O atoms is visible. The separate placements of Mg, Al, Si and O are given in Figure 3c–f.

**Figure 4:**
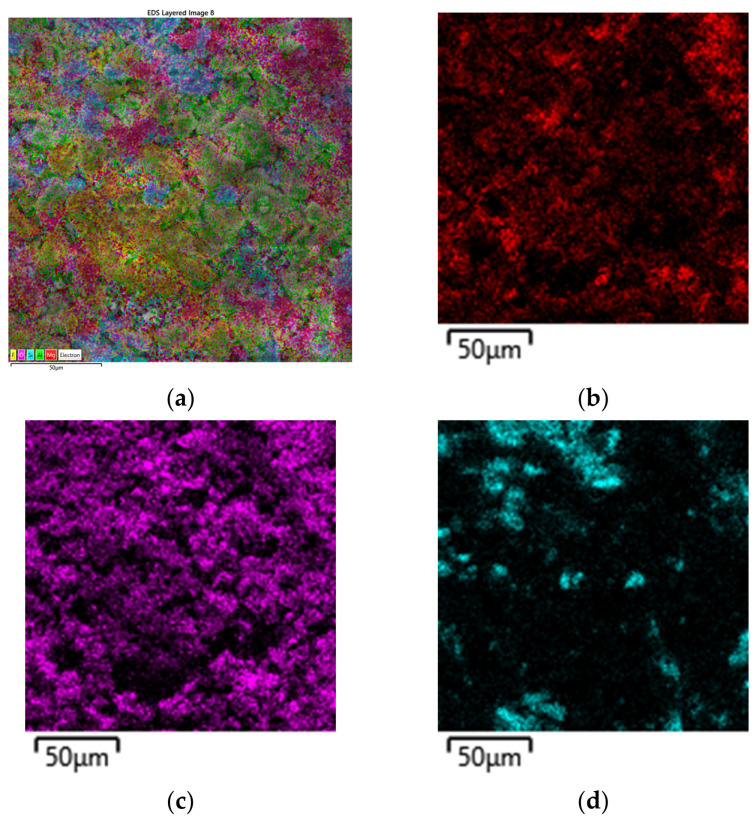

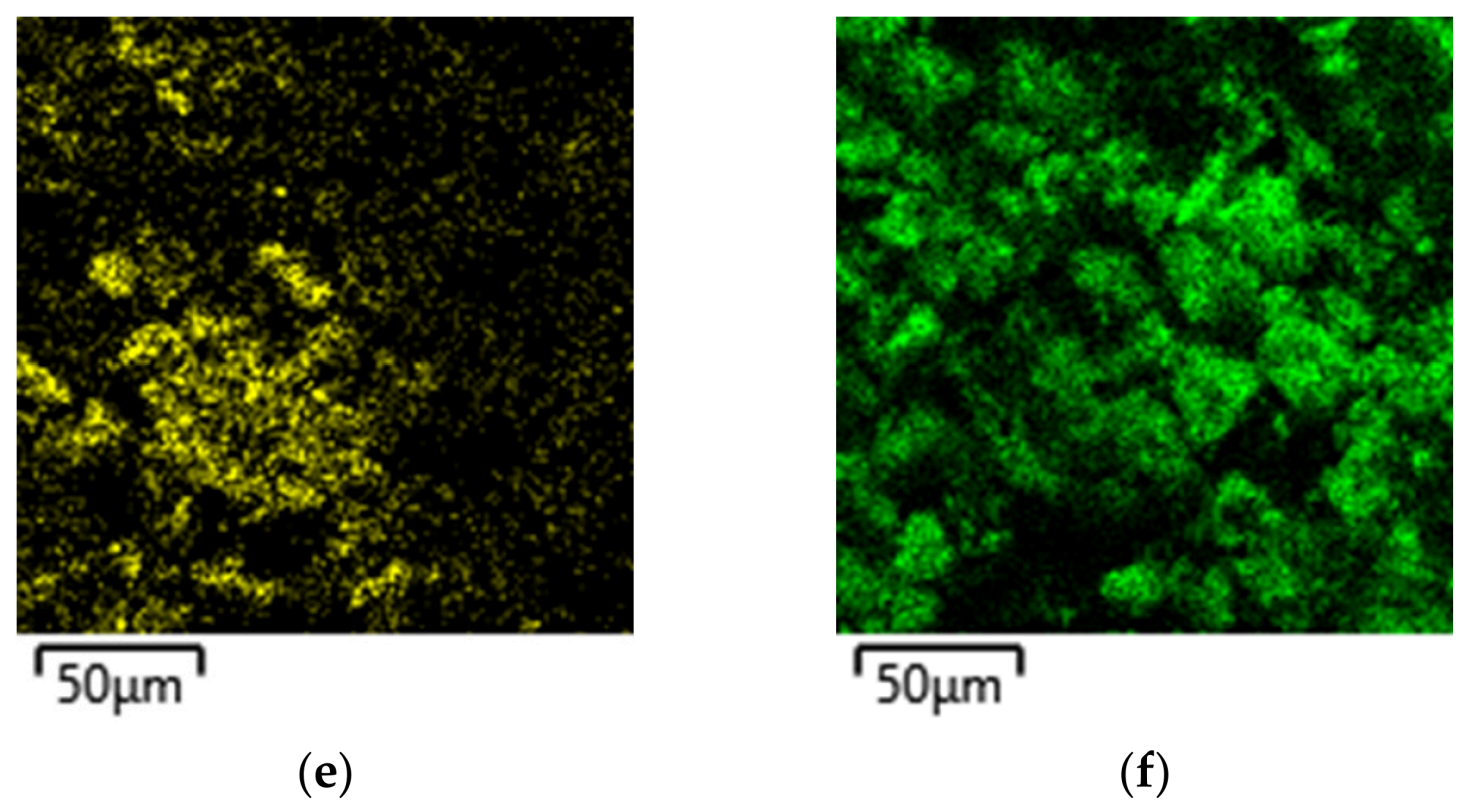
SEM micrograph of MgAl LDH@SiO_2_ particles after immersion in aqueous NaF solution: (**a**) composite image; (**b**–**f**) elemental distribution maps of Mg, O, Si, F, and Al. The micrographs confirm uniform deposition of Mg–Al hydroxide layers on silica cores and illustrate fluoride incorporation after exposure.

Samples of MgAl LDH@SiO_2_ after immersion in fluoride solution were tested using the SEM-EDS with the aim of confirming the presence of fluoride in the structure. The analysis confirmed that Mg–Al hydroxide layers were successfully deposited onto silica cores, forming uniform coatings rather than separate LDH aggregates (Figure 4). Elemental mapping by EDS verified the presence of Mg, Al, Si, O, and F, demonstrating measurable fluoride incorporation into the interlayer space (Figure 4b–f). Fluoride uptake was consistently higher in samples treated with ethanol–water mixtures compared to purely aqueous solutions. Detailed micrographs and EDS spectra are provided in the Supplementary Materials (Section S1), Figures S1–S6 [30].

EDS/SEM microstructural analysis confirmed the homogeneous deposition of Mg–Al hydroxide layers onto SiO_2_ particles, without the formation of separate LDH agglomerates. EDS mapping revealed the presence of Mg, Al, Si, and O, along with detectable fluoride incorporation after exposure to NaF solutions. Quantitative analysis highlighted significant differences in fluoride uptake between MgAl LDH@SiO_2_ and MgAl LDO@SiO_2_ specimens (Table 1). The highest fluoride content (14.7 wt.%, 15.5 at.%) was observed in MgAl LDO@SiO_2_ treated in ethanol–water solution, confirming the pronounced memory effect associated with oxide reconstruction into LDH. In contrast, MgAl LDH@SiO_2_ samples exhibited moderate fluoride incorporation (~4 wt.%), while specimens treated in pure aqueous solution showed minimal uptake (~2 wt.%) (Table 1). These findings demonstrate that ethanol–water mixtures facilitate ion transport and enhance the accessibility of active binding sites, thereby promoting superior fluoride incorporation compared to purely aqueous systems [31].

Taken together, these results confirm that MgAl LDO coatings are more reactive toward fluoride, particularly in ethanol–water mixtures, whereas MgAl LDH coatings remain structurally stable but limited in anion incorporation. This highlights solvent composition as a significant factor in controlling fluoride uptake and supports the use of MgAl LDOs as effective precursors for targeted anion capture.

BET analysis was performed on SiO_2_ particles and on MgAl LDH@SiO_2_ particles and they revealed that the added layers of LDH increased the specific surface of the material, enabling more open structure for ion exchange and memory effect. BET surface area analysis showed that silica exhibited a specific surface area of 6.98 m^2^ g^−1^ ± 0.66, while the MgAl LDH@SiO_2_ composite reached 19.1 m^2^ g^−1^ ± 0.86. The adsorption–desorption behavior and pore size distribution are shown in Figure S7 (Supplementary Materials).

**Figure 5:**
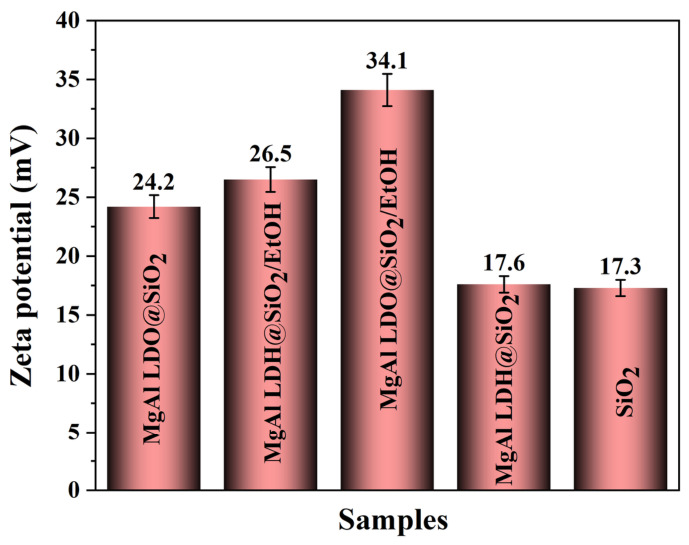
Zeta potential of particles after saturation in water, or ethanol–water solutions of fluoride.

The highest zeta potential is observed in the sample of MgAl LDO@SiO_2_ saturated with fluoride in ethanol–water solution (+34.1 mV). The high zeta potential means that the particles are stable in colloidal suspension and opens the structure and enables easier access to fluoride in the structure. The intermediate zeta potentials were observed in MgAl LDH@SiO_2_ saturated in ethanol–water solution (+26.5 mV), and MgAl LDO@SiO_2_ (+24.5 mV) saturated with fluoride in water solution. The lowest value of zeta potential is observed in the sample of MgAl LDH@SiO_2_ (+17.6 mV) saturated with fluoride in water solution. Interestingly, silica exhibited positive zeta potential, making it a relatively stable particle in the solution (Figure 5).

### 2.3. Fluoride Release Observation

Fluoride release from particles was tested in demineralized water, as this environment best reflects the intended application of the material. The neutral solvent was chosen also for the reason that MgAl LDH that is prepared in a high pH solution would possibly dissolve in a very acidic solution that usually is favorable for some materials’ ion exchange.

**Figure 6:**
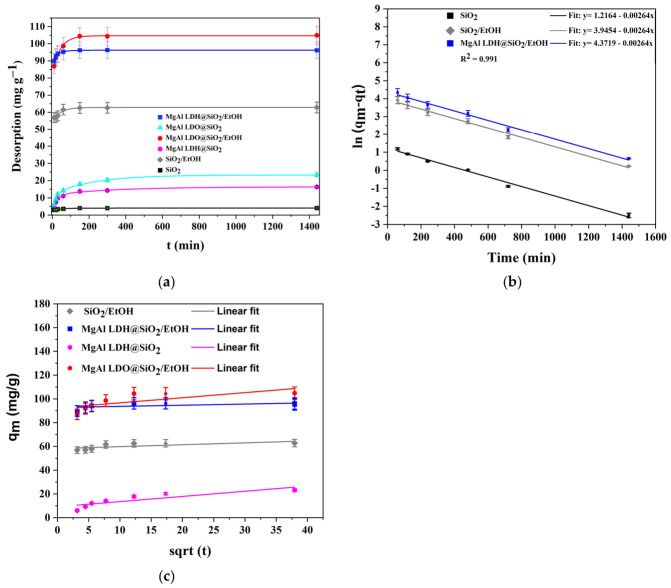
(**a**) Fluoride release from all specimens in demineralized water, with kinetic model fits, (**b**) First-order plots are shown for ion-exchange-dominated systems, and (**c**) Higuchi fits are emphasized for diffusion-controlled release.

Fluoride release was monitored by ion chromatography over 1440 min to track desorption dynamics in aqueous solution (Figure 6). Silica particles were also subjected to the same procedure and they served as controls to assess their intrinsic ability to retain and release fluoride. Results confirmed that pure silica shows negligible uptake and release, highlighting its limited role in the process. The observed fluoride release therefore originates primarily from the active MgAl LDH layer deposited on the silica surface during co-precipitation, rather than from the silica core itself.

To evaluate the release mechanisms, the experimental data were fitted to three common kinetic models: zero-order, first-order, and Higuchi diffusion. Zero-order assumes a constant release rate independent of concentration, first-order describes release governed by ion exchange with exponential decay, and the Higuchi model reflects diffusion-controlled release proportional to the square root of time. For each dataset, linearized plots were constructed (release vs. time, ln (qₘ − qₜ) vs. time, and release vs. √t), and correlation coefficients (R^2^) were calculated to determine the best fit. A pseudo-second-order kinetics model was also applied for mathematical rigor, although the three previous models are more sensitive for mechanistic interpretation.

Fluoride release experiments revealed clear differences in uptake capacity and governing mechanisms across the tested systems. Bare silica in water showed minimal release (~4 mg/g) with first-order kinetics (R^2^ ≈ 0.91), consistent with simple ion exchange at surface sites. In contrast, silica pre-treated in ethanol exhibited a much higher release (~63 mg/g) and followed Higuchi diffusion behavior (R^2^ ≈ 0.93), indicating deep incorporation facilitated by ethanol. MgAl LDH@SiO_2_ in water released ~16 mg/g and fit best to a first-order model (R^2^ ≈ 0.94), confirming ion exchange from LDH interlayers. When sorbed in ethanol, however, MgAl LDH@SiO_2_ reached ~96 mg/g and shifted to Higuchi diffusion kinetics (R^2^ ≈ 0.95), reflecting enhanced penetration of fluoride into the layered structure. MgAl LDO@SiO_2_ in water displayed higher release (~23 mg/g) with Higuchi behavior (R^2^ ≈ 0.94), attributable to the memory effect and partial reconstruction into LDH. Under high loading conditions, LDO@SiO_2_ reached ~105 mg/g and maintained diffusion-controlled release (R^2^ ≈ 0.95), demonstrating the strong influence of structural reconstruction on fluoride incorporation. Collectively, these results highlight the mechanistic progression from ion exchange in water to diffusion in ethanol-treated and LDO systems, underscoring the critical role of solvent environment and material structure in controlling fluoride uptake and release. First-order kinetics provided the best statistical fit in systems where fluoride was bound at accessible surface or interlayer sites, consistent with ion exchange processes. In contrast, ethanol treated and LDO systems exhibited deeper incorporation, where diffusion through reconstructed layers governed release. Although first-order models yielded high R^2^ values in these cases, mechanistic interpretation favors Higuchi diffusion as the dominant process.

The LDH layer undergoes ion exchange in aqueous solution, resulting in relatively rapid release of the incorporated fluoride. Consequently, the final fluoride concentration in solution is the lowest among all specimens. In contrast, the LDO samples exhibit higher fluoride concentrations compared to LDH in aqueous fluoride solution, confirming that the memory effect intensifies fluoride incorporation within the structure. The comparison of these two mechanisms has been previously studied [24], and the results obtained with composite particles validate those considerations. The “memory effect” involves recrystallization of LDO into LDH, during which anion incorporation is significantly more efficient than simple ion exchange.

The two specimens exposed to the water–ethanol fluoride solution exhibited higher fluoride content within the structure, as confirmed by EDS analysis, while the levels of fluoride released into the surrounding aqueous medium were also markedly higher. Ethanol appears to promote fluoride incorporation in both mechanisms (ion exchange and the memory effect), which includes the recrystallization of LDH from the layered LDO structure. The levels obtained are consistently higher for the memory effect compared to ion exchange, following the same trend observed in other samples.

The incorporation of ethanol during ion exchange and memory effect processes in MgAl LDH was observed to significantly enhance efficiency compared with purely aqueous systems. The level of released fluoride is approximately five times that obtained in water-only solution. The reduced polarity of ethanol lowered the degree of anion solvation, thereby facilitating their migration into the interlayer galleries. Concurrently, ethanol molecules intercalated between the layers, expanding the gallery spacing and improving ion mobility. This solvent environment stabilized the reconstructed LDH structure, enabling a more complete manifestation of the memory effect. In addition, ethanol suppressed particle aggregation, ensuring greater exposure of active sites for exchange [31]. Taken together, these effects—altered solvation dynamics, structural stabilization, and enhanced diffusion—account for the superior performance of ethanol-containing systems in promoting ion exchange and structural reconstruction [32].

These mechanistic differences not only enhance the understanding fluoride release in dental composites but also illustrate a broader principle in materials science: solvent-assisted ion incorporation and structural reconstruction can be harnessed for diverse controlled-release systems. The enhanced uptake observed in ethanol-treated specimens demonstrates how solvent polarity and interlayer dynamics can be tuned to regulate ion mobility, a concept that could be extended to drug delivery, nutrient release in agriculture, or catalytic processes. By situating the fluoride release behavior within this wider context, the present study highlights how LDH/LDO composites serve as a model system for multifunctional, adaptive materials [2].

**Figure 7:**
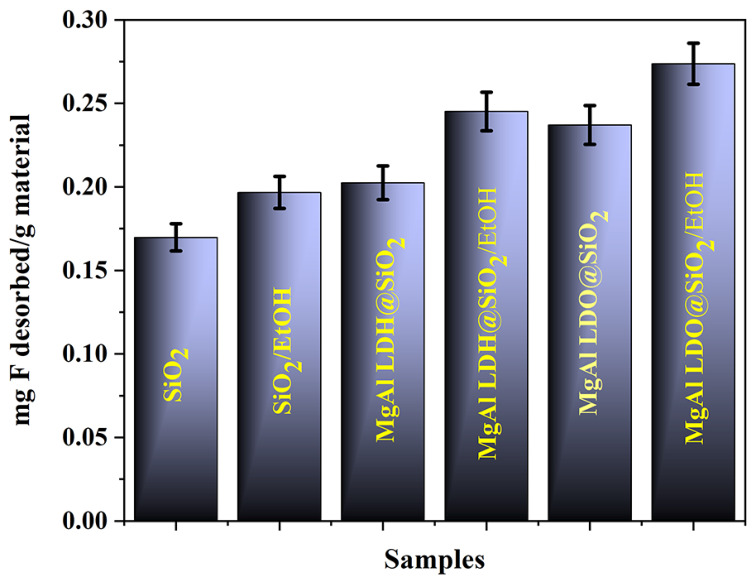
Fluoride release from composite tablets in demineralized water after 24 h. All particles were previously loaded in aqueous or ethanol–water fluoride solutions. Fluoride release reflects diffusion through the BisGMA/TEGDMA polymer matrix.

The time required to reach equilibrium concentration was relatively short, occurring within several hours for all specimens, indicating that the particles can adsorb and release fluoride rapidly. For potential dental applications, however, fluoride levels must be lower and the release rate slower than those observed in the free particle experiments. Since diffusion is inherently a gradual process, incorporation of such particles into a polymer matrix as functional fillers is expected to slow fluoride release, making them suitable candidates for sustained delivery of fluoride to dental tissue following intervention. In this context, the polymer selected was a combination of BisGMA (Bisphenol A glycidyl methacrylate) and TEGDMA (Triethylene glycol dimethacrylate)—photopolymerizing acrylate monomers long employed in adhesive dentistry. Small composite tablets containing 5 wt.% of the functional material were prepared, and fluoride release was monitored after 24 h under the same conditions as the individual particles. Figure 7 presents the fluoride levels observed after 1 day of exposure of the composite tablet to demineralized water.

Silica released the smallest amount of adsorbed fluoride into solution, with levels approximately 10 times lower than those observed for the free particles. The trend of fluoride release followed the same pattern as that of the individual fillers: the lowest levels were obtained from silica exposed to fluoride in aqueous solution, while slightly higher levels were observed for silica treated with the water–ethanol fluoride solution. In contrast, MgAl LDH@SiO_2_ composites released higher amounts of fluoride in demineralized water compared with pure silica, an increase attributed to fluoride incorporated within the interlayer space and subsequently released through the polymer matrix. The trend continued with MgAl LDO@SiO_2_ specimens, which exhibited even higher fluoride release, particularly when exposed to fluoride in the water–ethanol solution. This confirms that the memory effect provides superior incorporation of fluoride into the interlayer space of the functional filler. Importantly, all release levels from the composite tablets remained lower than those observed for the free particles and fall within the range suitable for use in dental materials with functional properties.

Beyond the immediate dental applications, the dual capacity of MgAl LDH@SiO_2_ composites to both capture and release fluoride underscores their multifunctional potential. In environmental contexts, these materials could act as sustainable sorbents for fluoride removal from contaminated water, while in biomedical settings they provide localized, sustained fluoride delivery to strengthen enamel. This convergence of remediation and healthcare functions reflects a growing paradigm in materials design, where fillers and reinforcements are expected to serve multiple roles. Future work should therefore explore mechanical performance in dental composites, biocompatibility under clinical conditions, and scalability of bio-silica sourcing, ensuring that the multifunctional promise of these materials translates into practical impact.

### 2.4. Mechanical and Surface Properties of the Prepared Acrylic Composites

To evaluate the mechanical characteristics of the composites, Vickers microhardness measurements were performed (see Table 2). Microhardness provides insight into the elastic behavior of the material and enables comparison across different formulations. The neat epoxy matrix exhibited a hardness of 125.75 MPa. Incorporation of unmodified silica resulted in only a slight increase (127.44 MPa), whereas all specimens containing 5 wt% of modified silica fillers showed markedly higher hardness values. These results indicate that the presence of silica improves hardness compared to the matrix, but the decisive factor is the surface modification of silica rather than the specific method of fluoride saturation. Differences among modified groups are relatively small, suggesting that the modification itself, rather than the fluoride treatment route, governs the hardness enhancement. Previous research indicated that similar particles, when incorporated into an acrylic composite, enhanced mechanical properties [10].

## 3. Materials and Methods

### 3.1. Materials Used in Synthesis

Details of all chemicals used in the experiments can be found in the Supplementary Materials, Section S3 [33].

### 3.2. Preparation of Particles

MgAl LDH@SiO_2_ composites were prepared by coprecipitation. Silica particles were dispersed in aqueous solutions of Mg(NO_3_)_2_·6H_2_O (0.45 mol) and Al(NO_3_)_3_·9H_2_O (0.15 mol), maintaining Mg:Al = 3:1. Precipitation was induced with 1 M NaOH to pH 10, followed by stirring for 10 h and ageing overnight. The precipitate was washed with deionised water and centrifuged (5000 rpm, three cycles) to neutral pH. Half of the LDH@SiO_2_ composite was calcined at 400 °C for 5 h, yielding MgAl LDO@SiO_2_. This temperature ensured complete dehydroxylation while avoiding spinel formation [34]. Stable mixed oxides with surface basicity have been reported at 400 °C [35]. A schematic representation of the preparation of LDH particles is shown in the Supplementary Materials (Section S4) in Figure S8.

### 3.3. Memory Effect Testing for MgALDO@SiO_2_

Fluoride adsorption was evaluated using two solutions: 0.15 mol/L aqueous NaF and a 0.15 mol/L 1:1 water/ethanol mixture. For each test, 0.5 g of sample was immersed in 80 mL of solution at 25 °C for 24 h, then filtered (blue filter paper) and dried at room temperature. Six samples were obtained for fluoride release testing and subsequent incorporation into dental composite materials.

Release from the dried powders was assessed by immersing 0.02 g of each sample in 20 mL of demineralized water at room temperature, with fluoride concentrations determined by ion chromatography (IC).

Dental composite tablets (~0.07 g) containing 5 wt.% of the functional material were prepared. Each tablet was immersed in 10 mL of demineralized water at 25 °C for 24 h. Fluoride concentration was measured and recalculated using Equation (1):

(1) $$q_{m}=\frac{C_{e}}{m_{t}}V_{s},$$

where q_m_ is the amount of fluoride released per gram of composite dental material (mg g^−1^), C_e_ is the equilibrium fluoride concentration in the solution after 24 h (mg L^−1^), m_t_ is the mass of the composite dental tablet (g), and V_s_ is the volume of liquid used for fluoride release (L).

All the kinetic models considered are presented in the Supplementary Materials Section S2 [36,37,38,39].

### 3.4. Preparation of Composite Materials

The composite matrix was formulated using BisGMA (bisphenol A glycidyl methacrylate, 49.5%), TEGDMA (triethylene glycol dimethacrylate, 49.5%), CQ (camphorquinone, 0.2%), and 4EDMAB (ethyl-4-dimethylaminobenzoate, 0.8%). To prepare the composites, 5 wt.% of SiO_2_, MgAl LDH@SiO_2_, or MgAl LDO@SiO_2_ particles were incorporated.

Specimens were produced in the form of discs with a diameter of 5 mm and a thickness of 2 mm, dimensions suitable for hardness evaluation. Both the matrix and the composite samples were cured under a UV lamp for 3 minutes per specimen (the lamp was sourced from Degussa, Germany, Frankfurt am Main, wavelength 400–500 nm, light intensity approximately 570 mW/cm^2^). The samples are labeled with Acr for matrix.

### 3.5. Material Characterization and Methods

Morphological features and elemental composition of MgAl LDH and MgAl LDO particles, prior to and following the adsorption process, were investigated using a MIRA3 TESCAN Field-Emission Scanning Electron Microscope (FE-SEM, TESCAN, Brno, Czech Republic) operating at 20 kV, in combination with Energy Dispersive X-ray Spectroscopy (EDS). The EDS measurements were carried out using an INCAx-act LN2-free Analytical Silicon Drift Detector, optimized for characteristic X-ray detection, and integrated with PentaFET^®^ Precision electronics and Aztec 4.3 software (Oxford Instruments, Abingdon, UK), connected to the TESCAN Mira3 XMU platform.

The crystalline structure of the synthesized particles was analyzed via X-ray Powder Diffraction (XRPD), employing an Ultima IV Rigaku diffractometer (Rigaku Corporation, Tokyo, Japan) configured with Bragg–Brentano geometry and CuKα radiation (λ = 1.5418 Å). Data acquisition was performed in step-scan mode over the 2θ range of 10–80°, with a step interval of 0.02° and a dwell time of 0.50 s per step.

Chemical bonding and functional group identification were conducted using Fourier Transform Infrared spectroscopy (FTIR). Spectral data were collected using a Nicolet™ iS™ 10 FT-IR Spectrometer (Thermo Scientific, Kwai Chung, Hong Kong), equipped with Smart iTR™ Attenuated Total Reflectance (ATR) accessories. Measurements were recorded in the spectral window of 4000–400 cm^−1^, with a resolution of 4 cm^−1^ and an average of 20 scans per sample.

XPS measurements were performed using a SPECS system equipped with an XP50M X-ray source (Focus 500) and a PHOIBOS 100 energy analyzer (SPECS Surface Nano Analysis GmbH, Berlin, Germany). Monochromatic Al Kα radiation (1486.74 eV) was applied at 12.5 kV and 16 mA. Samples were mounted on adhesive copper foil to ensure firm mechanical support and reliable electrical contact. The survey spectra (0–1000 eV BE) were collected with a pass energy of 40 eV, step size of 0.5 eV, and dwell time of 0.2 s. High-resolution spectra of individual core levels were acquired at 20 eV pass energy, 0.1 eV step size, and 2 s dwell time. Data acquisition was carried out using SpecsLab software (CasaXPS version 2.3.160ev52), while spectral deconvolution and analysis were performed with CasaXPS. A Shirley-type background was applied consistently to all spectra.

Nitrogen adsorption–desorption analysis was performed using a Micromeritics ASAP 2020 system (Micromeritics Instrument Corp., Norcross, GA, USA). Prior to measurement, the samples were degassed at 100 °C for 9 h under vacuum to remove adsorbed species. The specific surface area was determined by applying the Brunauer–Emmett–Teller (BET) method to the linear region of the adsorption isotherm. The total pore volume (V_total_) was obtained at a relative pressure of p/p_0_ = 0.998. Mesopore volume (V_meso_) was calculated from the desorption branch using the Barrett–Joyner–Halenda (BJH) method. The average pore diameter (Dsr) was derived from the BJH desorption data based on the relation 4V/S.

The zeta potential of the samples was determined using a Zetasizer Nano ZS equipped with a Multi-Purpose Titrator MPT-2 (Malvern Instruments Ltd., Malvern, UK). For the measurements, the dry powder samples were dispersed in distilled water at a concentration of 0.62 mg mL^−1^. The suspensions were then sonicated (Sonis 2 (G)T Series Desktop Ultrasonic Cleaner, Šentjernej, Slovenia) for 15 min at room temperature using a probe sonicator to ensure proper dispersion of the particles. Zeta potential measurements were performed using disposable polycarbonate capillary cells with gold-plated electrodes, with 850 μL of the sample analyzed.

Quantification of fluoride ions in aqueous samples was performed by ion chromatography, utilizing a Dionex IonPac AS14 analytical column (Thermo Fisher Scientific, Sunnyvale, CA, USA) (4 × 250 mm) coupled with a suppressed conductivity detector.

The microhardness characteristics of the fabricated composites were determined via the Vickers indentation method. A Leitz Kleinert Prüfer DURIMET I apparatus (Oberkochen, Germany) applied a constant force of 0.98 N with a 25 s holding time to obtain the hardness values.

## 4. Conclusions

Composite particles consisting of bioderived silica and MgAl LDH and MgAl LDO as surface modifications were used to capture fluoride from water and ethanol–water solutions, and the release of fluoride in demineralized water was observed. The incorporation of fluoride into those materials was obtained by ion exchange mechanism for MgAl LDH@SiO_2_ and as a memory effect mechanism in MgALDO@SiO_2_. The time required to reach equilibrium concentration during fluoride discharging process was relatively short, occurring within several hours for all specimens, indicating that the particles can adsorb and release fluoride rapidly. For potential dental applications, however, fluoride levels must be lower and the release rate slower than those observed in the free particle experiments. Since diffusion is inherently a gradual process, incorporation of such particles into a polymer matrix as reinforcing functional fillers slowed down fluoride release levels, making them suitable candidates for sustained delivery of fluoride to dental tissue following intervention. In this context, the composite incorporates polymer matrix made of BisGMA and TEGDMA—photopolymerizing acrylate monomers long employed in adhesive dentistry.

Notably, particles exposed to water–ethanol fluoride solutions incorporated higher fluoride concentrations and subsequently released greater amounts compared to those treated in pure water, with release levels approximately fivefold higher.

We acknowledge that the present study is limited by the relatively small sample size and the absence of long-term mechanical and biological testing. Nevertheless, the findings provide proof-of-concept evidence supporting the affinity and retention capacity of MAlLDH@SiO_2_ particles, and motivate further investigation into their performance under clinically and environmentally relevant conditions.

## Figures and Tables

**Table 1:** The results of the EDS analysis, elemental composition of tested powders after immersion in a fluoride solution.

Specimen	O, wt.%	Si, wt.%	Al, wt.%	Mg, wt.%	F, wt.%
MgAl LDH@SiO_2_	50.7	22.0	19.8	5.9	1.6
MgAl LDO@SiO_2_	52	16.7	19.2	10.6	2.1
MgAl LDH@SiO_2_/EtOH	53.6	14.4	13.6	14.2	4.2
MgAl LDO@SiO_2_/EtOH	51.6	11.4	11.6	11.2	14.2

**Table 2:** Vickers microhardness (HV, MPa) of epoxy composites containing functional particle reinforcements (mean ± SD, *n* = 8).

Samples	HV (MPa) ± SD
Acr (Matrix)	125.7 ± 8.99
Acr_SiO_2_	127.4 ± 9.23
Acr_SiO_2__LDH_Et	161.3 ± 4.35
Acr_SiO_2__LDH	166.5 ± 2.75
Acr_SiO_2__LDO_Et	156.9 ± 3.28
Acr_SiO_2__LDO	160.1 ± 3.91

## Data Availability

The data presented in this study are available on request from the corresponding author or co-authors. The data are not publicly available.

## Supplementary Materials

The following supporting information can be downloaded at: https://www.mdpi.com/article/10.3390/molecules31122180/s1, Figure S1: SEM micrograph of dried MgAl LDH@SiO_2_ particles after immersion in water NaF solution; (a) composite image; (b–e) elemental distribution maps of Mg, O, Si, F, and Al; Figure S2: EDS analysis of dried MgAl LDH@SiO_2_ particles after immersion in water NaF solution; Figure S3: SEM micrograph of dried MgAl LDH@SiO_2_ particles after immersion in water NaF solution; (a) composite image; (b–e) elemental distribution maps of Mg, O, Si, F, and Al; Figure S4: EDS analysis of dried MgAl LDO@SiO_2_ water particles after immersion in water solution; Figure S5: SEM micrograph of dried MgAl LDH@SiO_2_ particles after immersion in ethanol–water solution; (a) composite image; (b–e) elemental distribution maps of Mg, O, Si, F, and Al; Figure S6: EDS analysis of dried MgAl LDH@SiO_2_ particles after immersion in ethanol–water NaF solution; Figure S7: BET analysis of (a) SiO_2_ particles and (b) MgAl LDH@SiO_2_ particles; Table S1: Summary of all tested models for interpretation of fluoride release data; Table S2: Summary of preferred kinetic models and best-fit equations for fluoride release; Table S3: Summary of applied equations for kinetic analysis; Figure S8: A schematic representation of the preparation of MgAl LDH@SiO_2_ particles; Refs [30,31,33,36,37,38,39] are in the Supplementary Materials.

## Abbreviations

The following abbreviations are used in this manuscript:

LDH	Layered double hydroxide
LDO	Layered double oxide
BisGMA	Bisphenol A glycidyl methacrylate
TEGDMA	Triethylene glycol dimethacrylate
CQ	Camphorquinone
4EDMAB	Ethyl-4-dimethylaminobenzoate
FESEM	Field Emission Scanning Electron Microscopy
TEM	Transmission Electron Microscopy
EDS	Energy Dispersive X-ray Spectroscopy
BET	Brunauer–Emmett–Teller
XPS	X-ray Photoelectron Spectroscopy
FTIR	Fourier Transform Infrared spectroscopy
IC	Ion chromatography
XRPD	X-ray Powder Diffraction
