# Supplementary material for: Functional MgAl LDH@SiO2 Composites: Controlled Fluoride Delivery in Dentistry
Source: Molecules. 2026 Jun 22;31(12):2180. doi: 10.3390/molecules31122180 (PMC13304669; doi:10.3390/molecules31122180)
Supplement: Supplementary file 1 [file molecules-31-02180-s001.zip › molecules-4333616-supplementary.pdf]

Supplementary Materials

Multifunctional MgAl LDH@SiO_2 Composites: From Water Purification to Controlled Fluoride Delivery in Dentistry

Asma Alazreg[1], Marija M. Vuksanović[2*], Vladislav Tadić[3], Adela Egelja[2], Andrija Savić[2], Aleksandra Šaponjić[4], Radmila Jančić Heinemann[1]

[1] *University of Belgrade, Faculty of Technology and Metallurgy, Belgrade, Serbia; 20194028@estudent.tmf.bg.ac.rs (A.A) and radica@tmf.bg.ac.rs (R.J.H.)*

[2] *Department of Chemical Dynamics and Permanent Education, „VINČA" Institute of Nuclear Sciences - Na-tional Institute of the Republic of Serbia, University of Belgrade, Belgrade, Serbia; adela@vin.bg.ac.rs, (A.E.); savic@vin.bg.ac.rs, (A.S.); and marija.vuksanovic@vin.bg.ac.rs (M.M.V.)*

[3] *University of Belgrade, Institute of Chemistry, Technology and Metallurgy, National Institute of the Republic of Serbia, Belgrade, Serbia; vladatadic98@gmail.com (V.T)*

[4] *Department of Materials, „VINČA" Institute of Nuclear Sciences - National Institute of the Republic of Serbia, University of Belgrade, Belgrade, Serbia; acavuc@vin.bg.ac.rs (A.Š.)*

* Correspondence: radica@tmf.bg.ac.rs (R.J.H.)

S1. Detailed morphology images of samples

To evaluate the efficiency of composite particles in fluoride adsorption and release, MgAl layered double hydroxide (LDH) and layered double oxide (LDO) materials were synthesized and deposited onto SiO_2 particles derived from rice husk ash. This composite approach enabled direct comparison between LDH- and LDO-coated systems. Fluoride loading was performed under two solvent conditions—pure water and a water–ethanol mixture—to examine the influence of medium polarity on interlayer incorporation. The overarching goal was to promote fluoride intercalation within the layered structure and to assess the performance of each system under varied conditions.

The initial step in characterizing both the materials and the fluoride incorporation process involved examining particle morphology and elemental composition. This was achieved using Scanning Electron Microscopy (SEM) coupled with Energy Dispersive X-ray Spectroscopy (EDS). The Mg–Al hydroxide layers were successfully deposited onto the silica cores rather than forming separate LDH aggregates. The presence of surface hydroxyl groups on silica provides anchoring sites for LDH nucleation, which promotes thin and continuous layer formation. While we acknowledge that advanced imaging (e.g., TEM cross-sections) would provide more direct visualization of the core–shell architecture, the combination of elemental mapping and surface chemistry consideration supports the conclusion of uniform coating. The morphology and composition of MgAl LDH and LDO particles had been previously characterized, providing insight into the transformation mechanism whereby LDO rehydrates and reconstructs into the LDH structure, facilitating fluoride intercalation. This so-called memory effect has been shown to

outperform conventional ion exchange in terms of fluoride uptake efficiency [1]. In the present study, we focus on comparing two approaches: fluoride incorporation via LDH layers deposited on SiO_2 particles in aqueous solution, and fluoride uptake driven by the memory effect at the outer surface of the composite particles.

Figure S1 shows the elemental composition of the MgAl LDH deposited on SiO_2, indicating the presence of the main elements such as oxygen, magnesium, aluminum, silica and fluoride. The amount of fluoride is 1.6 wt.% but this proves that the ion is kept in the interlayer space in a measurable amount [2].

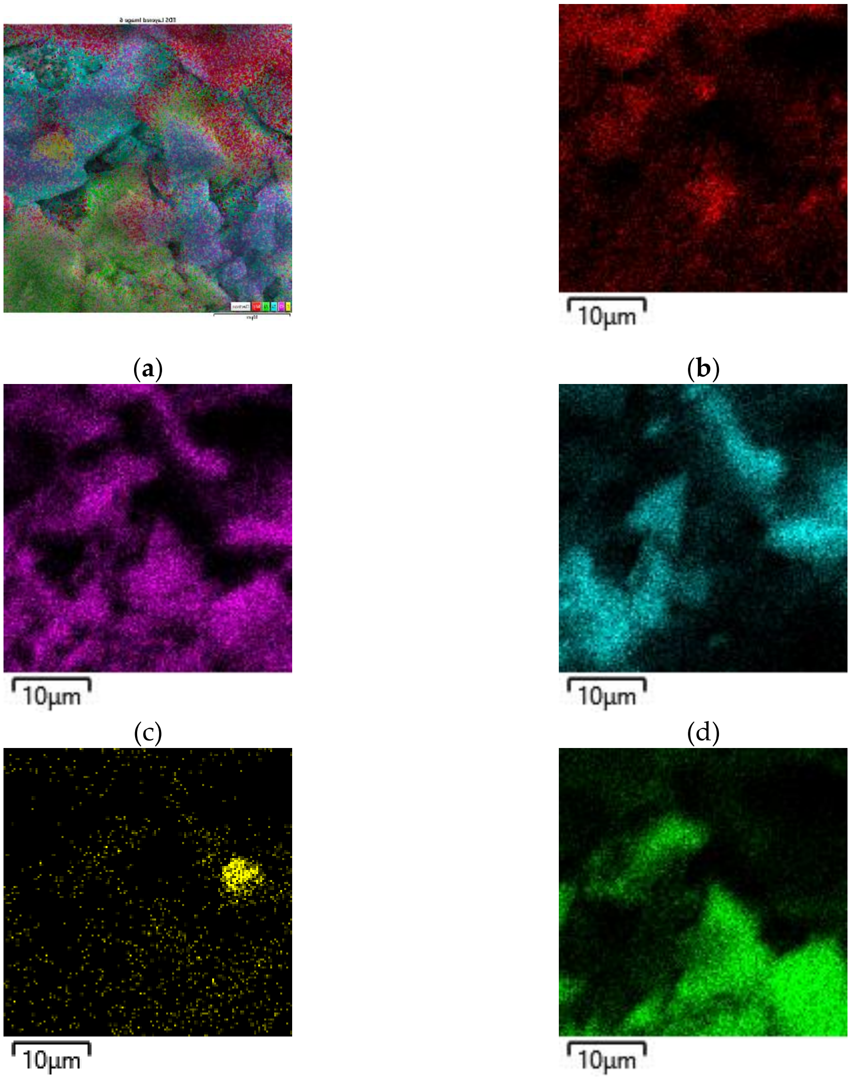

(**a**) (**b**) (c) (d) (e) (f)

Figure S1. SEM micrograph of dried MgAl LDH@SiO_2 particles after immersion in water NaF solution; (a) composite image; (b–e) elemental distribution maps of Mg, O, Si, F, and Al.

EDS analysis of dried MgAlLDH@SiO_2 water particles after immersion in water solution is presented in Figure S2.

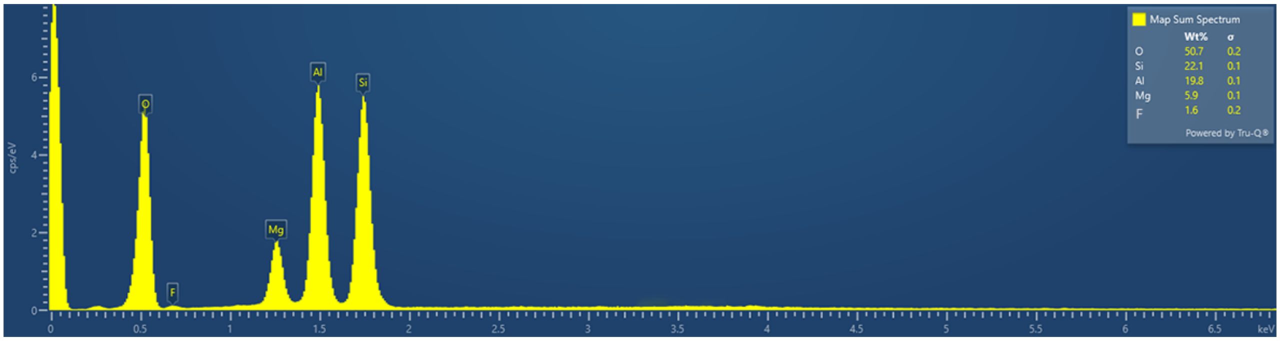

Figure S2. EDS analysis of dried MgAl LDH@SiO_2 particles after immersion in water NaF solution.

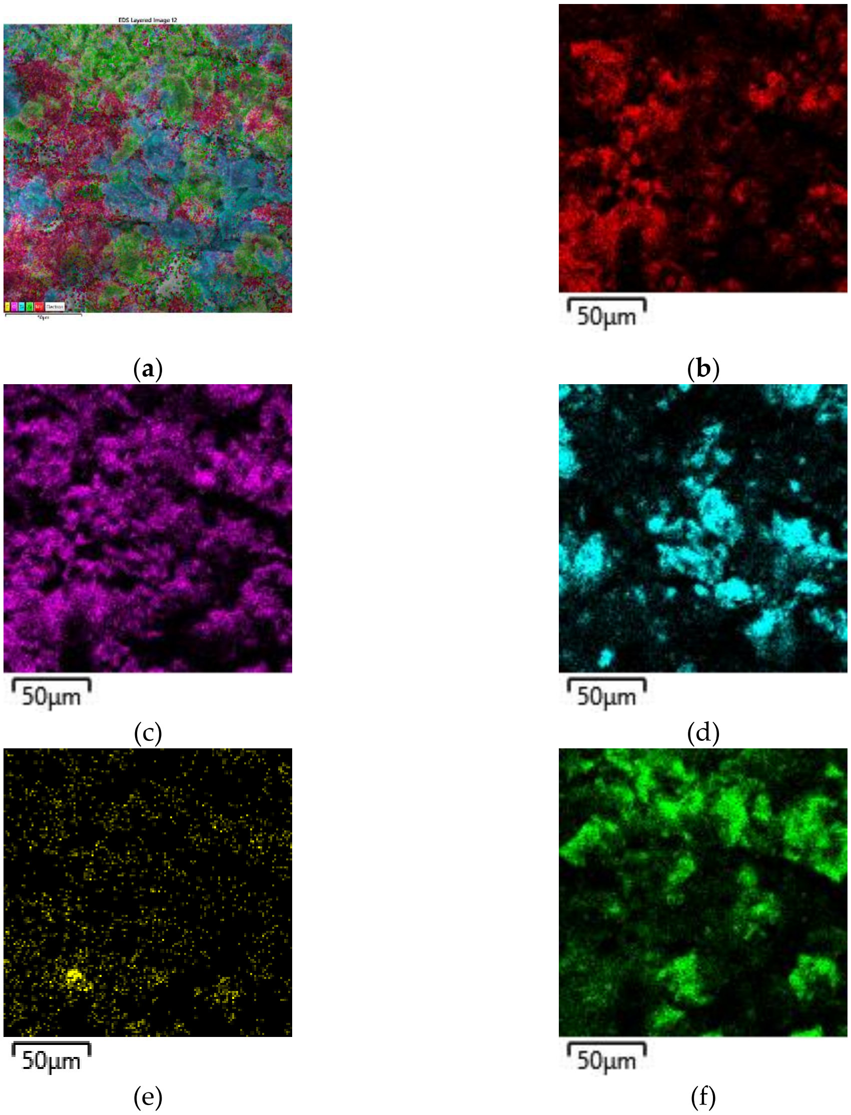

Figure S3. SEM micrograph of dried MgAl LDH@SiO_2 particles after immersion in water NaF solution; (a) composite image; (b–e) elemental distribution maps of Mg, O, Si, F, and Al.

EDS analysis of dried MgAl LDO@SiO_2 particles after immersion in water solution is presented in Figure S4.

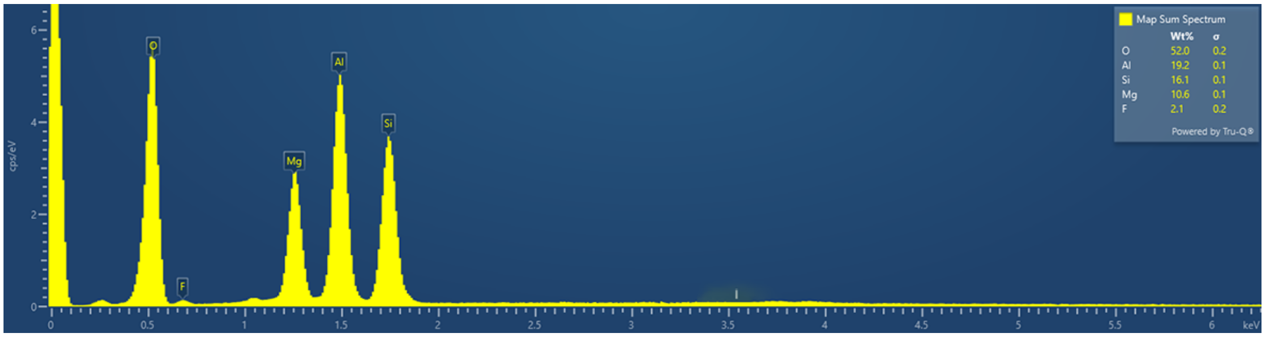

Figure S4. EDS analysis of dried MgAlLDO@SiO_2 water particles after immersion in water solution.

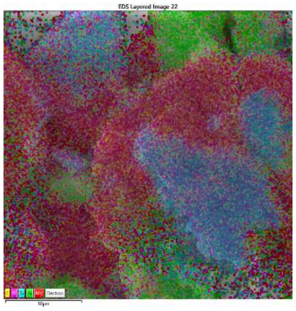

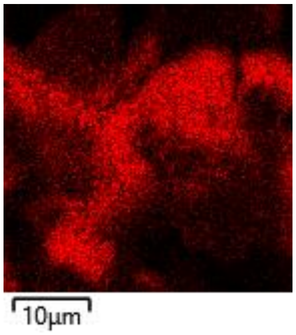

(**a**) (**b**)

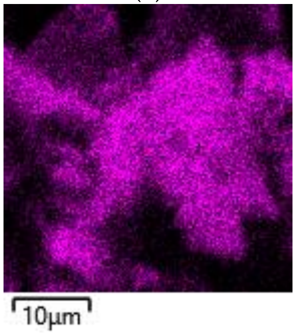

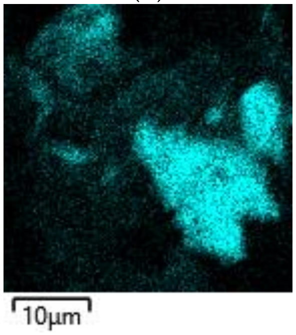

(c) (d)

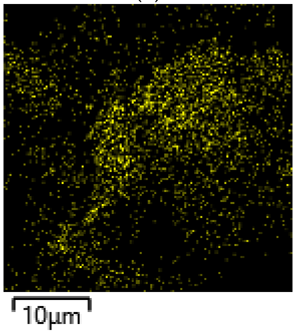

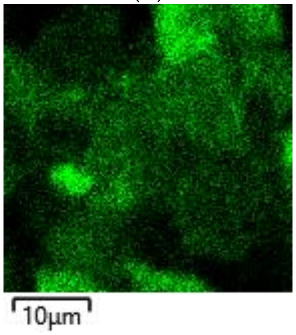

(e) (f)

Figure S5. SEM micrograph of dried MgAl LDH@SiO_2 particles after immersion in ethanol–water solution; (a) composite image; (b–e) elemental distribution maps of Mg, O, Si, F, and Al.

EDS analysis of dried MgAlLDH@SiO_2 water ethanol particles after immersion in water solution is presented in Figure S6.

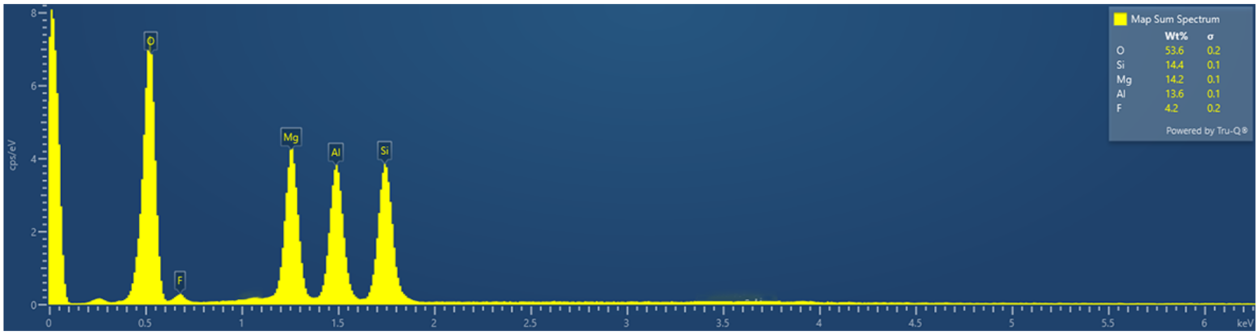

Figure S6. EDS analysis of dried MgAlLDH@SiO_2 particles after immersion in ethanol–water NaF solution.

Previously, the intercalation of fluoride was examined with the influence of memory effect that was proven to be beneficial in a water environment for the transformation of the LDO-LDH system. The maximum fluoride content measured in the MgAlLDO@SiO_2 immersed into the ethanol–water reached a fluoride content as high as 14.3 wt. % of fluoride, and 4.3 wt. % in water through the memory effect, while simple ion exchange reached only 2.1 wt. %. The results of these experiments were in the preparation of this research, so we only performed the examination of the first sample that was immersed in water to prove the bonding of the fluoride into the structure.

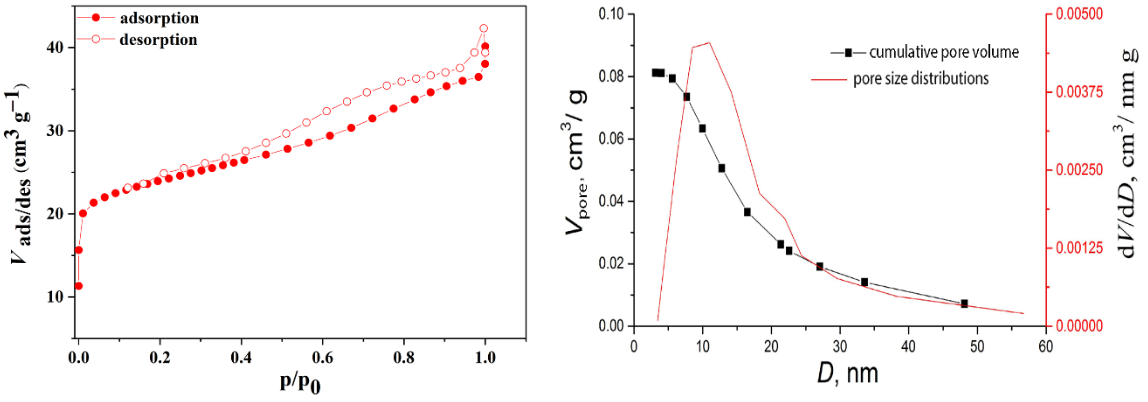

(**a**) (**b**)

Figure S7. BET analysis of (a) SiO2 particles and (b) MgAl LDH@SiO_2 particles.

S2. Analysis of kinetic models for obtained fluoride release data

Table S1. Summary of all tested models for interpretation of fluoride release data

Sample	q_m (mg/g)	Zero-order k	Zero-order R^2	First-order k	First-order R^2	Higuchi k	Higuchi R^2	Best Model
SiO_2	4.1	0.0007	0.76	0.0046	0.91	0.108	0.87	First-order
SiO_2/EtOH	62.8	0.0041	0.82	0.0032	0.89	0.52	0.93	Higuchi
MgAl LDH@SiO_2	16.3	0.0078	0.89	0.0049	0.94	0.39	0.91	First-order
MgAl LDH@SiO_2/EtOH	96.2	0.045	0.87	0.0091	0.91	3.12	0.95	Higuchi
MgAl LDO@SiO_2	23.3	0.012	0.87	0.0061	0.91	0.62	0.94	Higuchi
MgAl LDO@SiO_2/EtOH	105	0.066	0.88	0.012	0.91	4.85	0.95	Higuchi

Although first-order models produced high R^2 values across all systems, mechanistic interpretation based on fluoride incorporation depth and structural features supports diffusion-controlled release (Higuchi model) in ethanol-treated and LDO systems. Table S1 summarizes the preferred kinetic model, fitted rate constants, and regression coefficients for each sample.

Table S2. Summary of preferred kinetic models and best-fit equations for fluoride release

Sample	q_m (mg/g)	Preferred Model	Best-Fit Equation	R^2
SiO_2	4.1	First-order	$\ln(q_m - q_t) = \ln(q_m) - 0.0046 \cdot t$	0.91
SiO_2/EtOH	62.8	Higuchi	$q_t = 0.52 \cdot \sqrt{t}$	0.93
MgAlLDH@SiO_2	16.3	First-order	$\ln(q_m - q_t) = \ln(q_m) - 0.0049 \cdot t$	0.94
MgAlLDH@SiO_2/EtOH	96.2	Higuchi	$q_t = 3.12 \cdot \sqrt{t}$	0.95
MgAlLDO@SiO_2	23.3	Higuchi	$q_t = 0.62 \cdot \sqrt{t}$	0.94
MgAlLDO@SiO_2/EtOH	105.0	Higuchi	$q_t = 4.85 \cdot \sqrt{t}$	0.95

Table S3. Summary of applied equations for kinetic analysis

Model	Equation	Mechanism Type
**First-order model** [4]	$\ln(q_m - q_t) = \ln(q_m) - k_1 t$	Ion-exchange processes (surface/interlayer)
**Higuchi diffusion** [5]	$q_t = k_H$	Diffusion-controlled release (ethanol/LDO)
**Zero-order model** [6]:	$q_t = k_0 t$	Constant release rate (polymer composites)
**Pseudo-second-order** [7]	$\frac{t}{q_t} = \frac{1}{k_2 q_e^2} + \frac{t}{q_e}$	Chemisorption processes

For each dataset, linearized plots were generated (e.g., $\ln(q_m - q_t)$ vs. time, q_t vs. √t, q_t vs. time, and t/q_t vs. time), and regression coefficients (R^2) were calculated to assess the goodness of fit. The best-fit model was selected based on both statistical correlation and mechanistic plausibility. While pseudo-second-order kinetics produced high R^2 values, mechanistic interpretation favored first-order ion exchange for silica in water and Higuchi diffusion for LDH and LDO systems in ethanol or under high loading, where (q_m) is the maximum release capacity, (q_t) is the release at time (t), and (k_1) is the rate constant for the first-order mechanism, (k_H) is the constant for the Higuchi model, (k_0) is the constant for the zero-order model, and (k_2) is the constant for pseudo-second-order kinetics.

- **First-order model** [4]:
- [\ln(q_m - q_t) = \ln(q_m) - k_1 t]
  where (q_m) is the maximum release capacity, (q_t) the release at time (t), and (k_1) the rate constant. This model is commonly used to describe ion exchange processes.
- **Higuchi diffusion model** [5]:
  [q_t = k_H \cdot t^{0.5}]
  where (k_H) is the Higuchi constant. This model assumes diffusion-controlled release and is widely applied to systems where solvent-mediated transport or memory effect reconstruction occurs.
- **Zero-order model** [6]:
  [q_t = k_0 \cdot t]
  where (k_0) is the release rate constant. This model describes constant release rates, often observed in polymer–matrix composites.
- **Pseudo-second-order model** [7]:
  [\frac{t}{q_t} = \frac{1}{k_2 q_e^2} + \frac{t}{q_e}]
  where (q_e) is the equilibrium release and (k_2) the rate constant. This model is typically associated with chemisorption processes.

S3. Materials

Magnesium nitrate ($Mg(NO_3)_2 \cdot 6H_2O$) and aluminium nitrate ($Al(NO_3)_3 \cdot 9H_2O$) (Sigma Aldrich) were employed as precursor materials for the synthesis of magnesium–aluminium layered double hydroxide (MgAl LDH). Nitrate salts were chosen due to their suitability for ion exchange investigations

[3]. Subsequently, a sodium fluoride solution (Merck) was used to examine the ion exchange properties of the MgAl LDH sample.

The composite matrix consisted of BisGMA (bisphenol A glycidyl methacrylate, 49.5%), TEGDMA (triethylene glycol dimethacrylate), camphorquinone (0.2%), and 4EDMAB (ethyl-4-dimethylaminobenzoate, 0.8%), all obtained from Sigma-Aldrich.

S4. Preparation of particles

A schematic representation of the preparation of particles from the precursor as well as the production of LDH is shown in Figure S8.

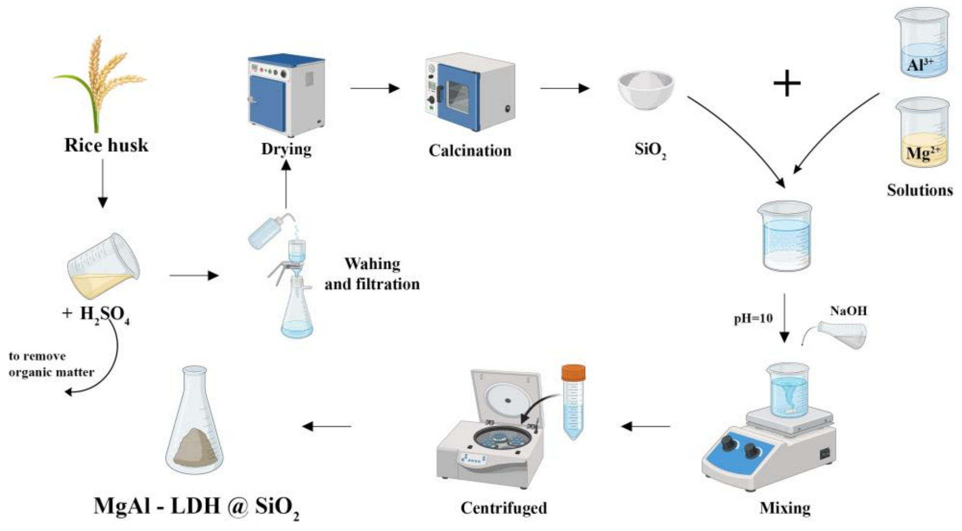

Figure S8. A schematic representation of the preparation of MgAl LDH@SiO_2 particles.

Fluoride desorption profiles were analyzed using several established kinetic models to identify the dominant release mechanisms. The applied are summarized in Table S2.

References

1. Alazreg, A.; Tadić, V.; Egelja, A.; Savić, A.; Šaponjić, A.; Vuksanović, M.M.; Heinemann, R.J. Memory Effect of Double Oxides Compared to Simple Ion Exchange for Controlled Fluoride Ion Capture and Release. *Materials (Basel).* **2025**, *18*, 162, doi:10.3390/ma18010162.

2. Pinthong, P.; Praserthdam, P.; Jongsomjit, B. Effect of Calcination Temperature on Mg-Al Layered Double Hydroxides (LDH) as Promising Catalysts in Oxidative Dehydrogenation of Ethanol to Acetaldehyde. *J. Oleo Sci.* **2019**, *68*, 95–102, doi:10.5650/jos.ess18177.

3. Leont'eva, N.N.; Cherepanova, S. V.; Stepanova, L.N.; Drozdov, V.A.; Lavrenov, A. V. Structural Aspects of "Memory Effect" for MgGa LDHs: New Data Obtained by Simulation of XRD Patterns for 1D Disordered Crystals. *Crystals* **2022**, *12*, 629, doi:10.3390/cryst12050629.

4. S. Lagergren Kungliga Svenska Vetenskapsakademiens. *Handlingar* **1898**, *24*, 1–39.

5. Talevi, A.; Ruiz, M.E. Higuchi Model. In *The ADME Encyclopedia*; Springer International Publishing: Cham, 2021; pp. 1–5.

6. Geçgel, Ü.; Üner, O.; Gökara, G.; Bayrak, Y. Adsorption of Cationic Dyes on Activated Carbon Obtained

from Waste Elaeagnus Stone. *Adsorpt. Sci. Technol.* **2016**, *34*, 512–525, doi:10.1177/0263617416669727.

7. Ho, Y..; McKay, G. Pseudo-Second Order Model for Sorption Processes. *Process Biochem.* **1999**, *34*, 451–465, doi:10.1016/S0032-9592(98)00112-5.
